# Impact on Macrolide Resistance of Genetic Diversity of Mycobacterium abscessus Species

**DOI:** 10.1128/spectrum.02749-22

**Published:** 2022-11-23

**Authors:** Bor-En Jong, Ting-Shu Wu, Nan-Yu Chen, Cheng-How Yang, Chin-Chung Shu, Lih-Shinn Wang, Tsu-Lan Wu, Jang-Jhih Lu, Cheng-Hsun Chiu, Hsin-Chih Lai, Wen-Hung Chung

**Affiliations:** a Division of Urology, Taipei Tzu Chi Hospital, Buddhist Tzu Chi Medical Foundation, New Taipei City, Taiwan; b Division of Infectious Diseases, Department of Internal Medicine, Linkou Chang Gung Memorial Hospital, Taoyuan City, Taiwan; c School of Medicine, Chang Gung University, Taoyuan City, Taiwan; d Department of Internal Medicine, National Taiwan University Hospital, National Taiwan University College of Medicine, Taipei, Taiwan; e Section of Infectious Diseases, Department of Internal Medicine, Hualien Tzu Chi Hospital, Hualien, Taiwan; f Department of Laboratory Medicine, Linkou Chang Gung Memorial Hospital, Taoyuan City, Taiwan; g Department of Medical Biotechnology and Laboratory Medicine, College of Medicine, Chang Gung University, Taoyuan City, Taiwan; h Department of Pediatrics, Linkou Chang Gung Memorial Hospital, Taoyuan City, Taiwan; i Department of Dermatology, Linkou Chang Gung Memorial Hospital, Taoyuan City, Taiwan; Tainan Hospital, Department of Health, Executive Yuan

**Keywords:** macrolide resistance, phylogenetic analysis, genetic mosaicism, antimycobacterial agents

## Abstract

Our previous study identified that the Mycobacterium abscessus subsp. *abscessus* T28 sequevar does not fully represent inducible macrolide resistance. Thus, we initiated a correlation study between genotypes and phenotypes. In total, 75 isolates from patients with skin and soft tissue infections were enrolled in the study. These strains were tested against 11 antimycobacterial agents using Sensitire RAPMYCO plates and the CLSI-recommended broth microdilution method. In order to analyze *erm*(41) and partial *hsp65*, *rpoB*, *secA1*, and *rrl* genes, bacterial genomic DNA was extracted from bacteria. The MEGA X software was used for phylogenetic analyses. The most active agents against most M. abscessus species were amikacin and tigecycline. Clarithromycin was effective toward M. abscessus subsp. *massiliense* and nearly all M. abscessus subsp. *abscessus* C28 sequevars. Two varieties of M. abscessus subsp. *abscessus* T28 sequevars did not represent inducible macrolide resistance. Most M. abscessus species showed intermediate susceptibility to cefoxitin and imipenem. Six additional agents were less effective against M. abscessus species. Following phylogenetic analyses, two outliers of M. abscessus subsp. *abscessus* T28 sequevars seem to represent no inducible macrolide resistance. In addition, we discovered genetic mosaicism of *hsp65*, *rpoB*, and *secA1* in M. abscessus species was common. T28 sequevars of M. abscessus subsp. *abscessus* do not fully represent inducible macrolide resistance. The outlier of *erm*(41) phylogeny of the M. abscessus subsp. *abscessus* T28 sequevar is possibly due to macrolide susceptibility. Evaluation of the antimicrobial susceptibility of M. abscessus species is a reliable tool for assisting physicians in selecting the most effective antimycobacterial agent(s).

**IMPORTANCE** Macrolides are the mainstays of the antimycobacterial regimens against Mycobacterium abscessus species (formerly Mycobacterium abscessus complex). *erm*(41) confers inducible macrolide resistance for M. abscessus subsp. *bolletii* strains, and the majority of M. abscessus subsp. *abscessus* T28 sequevars. Furthermore, the acquired macrolide resistance of M. abscessus species is due to a point mutation in *rrl*. However, not all M. abscessus subsp. *abscessus* T28 sequevars have inducible macrolide resistance. Exploration of the mechanism of macrolide resistance requires an understanding of genetic diversity. The genetic mosaicism of the *erm*(41), *rpoB*, *hsp65*, and *secA1* genes within three subspecies of M. abscessus species is not uncommon. The T28 sequevar of *erm*(41) confers inducible macrolide resistance to the genetic mosaic strain. The development of new anti-M. abscessus species infection overcoming inducible macrolide resistance and/or acquired macrolide resistance is a crucial issue.

## INTRODUCTION

The Mycobacterium abscessus species (formerly M. abscessus complex [MABC]) belongs to the rapidly growing mycobacteria (RGM). The prevalence of RGM infection has increased in recent years (1, 2), making MABC an important pathogen causing pulmonary infection, skin and soft tissue infection, bone and joint infection, central nervous system infection, bloodstream infection, and other infections in humans (3–8). Moreover, MABC tends to be multidrug resistant, which makes such infections difficult to treat (9).

Macrolide-based combination regimens were recommended by the American Thoracic Society/European Respiratory Society/European Society of Clinical Microbiology and Infectious Diseases/Infectious Diseases Society of America (ATS/ERS/ESCMID/IDSA) for both pulmonary and extrapulmonary infections (10, 11). The most recent nontuberculous mycobacterial (NTM) pulmonary disease treatment guidelines recommend a macrolide-based multidrug regimen containing at least three active antibiotics (11). However, most MABC isolates exhibit inducible and/or acquired resistance to macrolide antibiotics, making the treatment of MABC infections a crucial issue. In a retrospective cohort study by Sfeir et al. (12), macrolide resistance was identified as a risk factor for early treatment failure of MABC infection.

Current taxonomy divides MABC into three subspecies: M. abscessus subsp. *abscessus*, M. abscessus subsp. *massiliense*, and M. abscessus subsp. *bolletii* (13). Moreover, *erm*(41) sequences of the three subspecies differ, resulting in distinct macrolide susceptibility patterns. The gene *erm*(41) confers the ability to produce erythromycin ribosome methylase (Erm) on most MABC isolates. Erm decreases macrolide affinity to the ribosome exit tunnel by methylating the A2058 (corresponding to Escherichia coli numbering) nucleotide of the 23S rRNA gene (14). Approximately 80% of MABC isolates containing this inducible *erm*(41) gene, which may result in poor treatment outcomes (4). M. abscessus subsp. *massiliense* isolates have a truncated *erm*(41) gene, which renders them susceptible to macrolides and improves treatment (4). M. abscessus subsp. *bolletii* and ~80% of M. abscessus subsp. *abscessus* isolates contain a functional *erm*(41) gene that induces macrolide resistance (15). Although the exact mechanism is unknown, Nash et al. (16) discovered that isolates containing the *erm*(41) T28 sequevar possess the inducible macrolide resistance *erm*(41) gene, whereas isolates containing the C28 sequevar do not. In a previous study by Lee et al. (5), the relationship between the genotype of the *erm*(41) T28 polymorphism and its corresponding phenotype (inducible macrolide resistance) corroborated the findings of Nash et al. (16) in the majority of instances. Intriguingly, Lee et al. (5) identified two M. abscessus subsp. *abscessus erm*(41) T28 sequevars susceptible to clarithromycin (CLA) from the 3rd day of incubation (the initial reading time [IRT]) (MICs of 0.25 and 0.25 μg/mL) to the 14th day of incubation (the late reading time [LRT]) (MICs of 0.25 and 0.5 μg/mL) that lacked inducible macrolide resistance. In order to investigate the discrepancy between the genotype and phenotype of the *erm*(41) gene, additional research is required. In this study, phylogenetic analyses of *erm*(41), *hsp65*, *rpoB*, and *secA1* genes were performed on 75 isolates collected between 1 August 2012 and 31 March 2018, and the relationship between genotype and phenotype will be discussed.

## RESULTS

### Antimicrobial susceptibility testing.

The results of *in vitro* antimicrobial susceptibility testing (AST) of MABC are shown in Table 1. M. abscessus subsp. *abscessus* demonstrated high levels of resistance to ciprofloxacin (CIP) (33/35 [94.29%]), doxycycline (DOX) (35/35 [100%]), linezolid (LZD) (30/35 [85.71%]), minocycline (MIN) (34/35 [97.14%]), moxifloxacin (MXF) (34/35 [97.14%]), and trimethoprim-sulfamethoxazole (SXT) (33/35 [94.29%]). M. abscessus subsp. *massiliense* was also highly resistant to the antibiotics listed above: CIP (36/39 [92.31%]), DOX (38/39 [97.44%]), LZD (28/39 [71.79%]), MIN (38/39 [97.44%]), MXF (38/39 [97.44%]), and SXT (35/39, 89.74%). Both M. abscessus subsp. *abscessus* and M. abscessus subsp. *massiliense* were resistant or intermediately susceptible to cefoxitin (FOX) with 88.57% (31/35) and 71.79% (28/39), respectively. Imipenem (IMI) was less susceptible against both M. abscessus subsp. *abscessus* (0%) and M. abscessus subsp. *massiliense* (1/39 [2.56%]). Amikacin (AMK) and tigecycline (TGC) performed admirably against M. abscessus subsp. *abscessus* and M. abscessus subsp. *massiliense*. M. abscessus subsp. *abscessus* and M. abscessus subsp. *massiliense* had AMK susceptibility rates of 94.29% (33/35) and 87.18% (34/39), respectively. The MIC_50_ and MIC_90_ values of TGC for M. abscessus subsp. *abscessus* were 0.5 and 2 mg/L, while the MIC_50_ and MIC_90_ values for M. abscessus subsp. *massiliense* were 0.5 and 1 mg/L, respectively.

**TABLE 1:** Results from testing of antimicrobial susceptibility of 74 Mycobacterium abscessus species isolates to 10 antibiotics with susceptible, intermediate, and resistant MIC breakpoints according to CLSI recommendations^*a*^

Subspecies and drug^*b*^	MIC (μg/mL)			Susceptibility, *n* (%)^*c*^		
	MIC_50_	MIC_90_	MIC range	S	I	R
M. abscessus subsp. *abscessus* (*n* = 35)						
Amikacin	16	32	8 to >64	30 (85.7)	3 (8.6)	2 (5.7)
Cefoxitin	64	128	32 to 128	0	31 (88.6)	4 (11.4)
Ciprofloxacin	>4	>4	1 to >4	1 (2.9)	1 (2.9)	33 (94.3)
Clarithromycin						
IRT	0.5	4	0.06 to 4	30 (85.7)	5 (14.3)	0
LRT	>16	>16	0.12 to >16	10 (28.6)	3 (8.6)	22 (62.9)
Doxycycline	>16	>16	16 to >16	0	0	35 (100)
Imipenem	16	64	8 to >64	0	20 (57.1)	15 (42.9)
Linezolid	>32	>32	8 to >32	1 (2.9)	4 (11.4)	30 (85.7)
Minocycline	>8	>8	4 to >8	0	1 (2.9)	34 (97.1)
Moxifloxacin	>8	>8	2 to >8	0	1 (2.9)	34 (97.1)
Trimethoprim-sulfamethoxazole	>8/152	>8/152	2/38 to >8/152	2 (5.7)	0	33 (94.3)
M. abscessus subsp. *massiliense* (*n* = 39)						
Amikacin	16	32	8 to >64	34 (87.2)	3 (7.7)	2 (5.1)
Cefoxitin	64	128	32 to >128	0	28 (71.8)	11 (28.2)
Ciprofloxacin	>4	>4	2 to >4	0	2 (5.1)	37 (94.9)
Clarithromycin						
IRT	0.25	0.5	0.06 to >16	38 (97.4)	0	1 (2.6)
LRT	0.25	1	0.06 to >16	37 (94.9)	1 (2.6)	1 (2.6)
Doxycycline	>16	>16	4 to >16	0	1 (2.6)	38 (97.4)
Imipenem	16	32	4 to 64	1 (2.6)	21 (53.8)	17 (43.6)
Linezolid	>32	>32	2 to >32	7 (17.9)	4 (10.3)	28 (71.8)
Minocycline	>8	>8	2 to >8	0	1 (2.6)	38 (97.4)
Moxifloxacin	>8	>8	2 to >8	0	1 (2.6)	38 (97.4)
Trimethoprim-sulfamethoxazole	8/152	>8/152	0.5/9.5 to >8/152	4 (10.3)		35 (89.7)

a The results for the single Mycobacterium abscessus subsp. *bolletii* isolate are not displayed in the table.

b IRT, initial reading time; LRT, late reading time.

c By MIC: S, susceptible; I, intermediate susceptible; R, resistant.

Among the 35 M. abscessus subsp. *abscessus* isolates, there were 30 (85.7%) susceptible to clarithromycin (CLA) at the IRT and 5 (14.3%) isolates intermediate to CLA at the IRT. Among the 30 M. abscessus subsp. *abscessus* isolates susceptible to CLA at the IRT, 10 isolates remained susceptible to CLA at the LRT, three isolates became intermediately susceptible to CLA, and 17 isolates presented inducible macrolide resistance at the LRT. Five of the 35 isolates with intermediate CLA MICs during the IRT exhibited inducible macrolide resistance during the LRT. Twenty-four of the 35 M. abscessus subsp. *abscessus* isolates were T28 sequevars. According to a previous study by Nash et al. (16), 22 T28 sequevars exhibited inducible macrolide resistance. The remaining two T28 sequevars (MIS128 and MIS219) remained CLA susceptible at both IRT and LRT. Seventeen (77.3%) of the 22 isolates exhibiting inducible macrolide resistance were susceptible to AMK.

Similarly, 97.4% (38/39) of our M. abscessus subsp. *massiliense* isolates were susceptible to CLA at the IRT, and 94.9% (37/39) were susceptible to macrolides at the LRT. At the IRT, MIS251 was susceptible to CLA, but at the LRT, it was intermediate. MIS127 was the only M. abscessus subsp. *massiliense* isolate that exhibited resistance to CLA at both the IRT and LRT. Subsequently, this isolate was found to have a point mutation in *rrl* with an A2059G mutation in its 23S rRNA. The CLA MICs of M. abscessus subsp. *massiliense* were still lower than those of M. abscessus subsp. *abscessus* at the IRT (*P* = 0.0001).

Unsurprisingly, the solitary M. abscessus subsp. *bolletii* isolate contained a functional *erm*(41) sequevar. It exhibited susceptibility to AMK, intermediate susceptibility to FOX, and resistance to CIP, DOX, IMI, LZD, MIN, MXF, and SXT.

### Relationship between *erm*(41) point mutations and clarithromycin MICs.

Table 2 displays three point mutation patterns of the *erm*(41) gene and one point mutation of *rrl* of MABC. Three point mutation patterns of the *erm*(41) included nonsense mutation (C199T), a missense mutation (T28C, G76A, G158A, A238G, and C419T), and silent mutation (A120G, T159C, G168C, G255A, G279T, A330C, and T336C). T28C, G158A, and C199T of the *erm*(41) gene and A2059G of *rrl* were different from the wild phenotypes among the four mutation patterns.

**TABLE 2:** Relationship between point mutations of the *erm*(41) and *rrl* genes and clarithromycin susceptibility in Mycobacterium abscessus species

Mutation (*n*)	Clarithromycin MIC (μg/mL) at^*a*^:					
	IRT			LRT		
	MIC_50_	MIC_90_	MIC range	MIC_50_	MIC_90_	MIC range
*erm*(41)						
Nonsense mutation C199T^*b*^ (1)			0.12			0.12
Missense mutations						
T28C^*c*^ (11)	0.25	0.25	0.12 to 0.5	2	4	0.12 to 4
G76A^*d*^ (1)			4			>16
G158A^*e*^ (1)			0.25			0.5
A238G^*f*^ (21)	0.25	4	0.06 to 4	4	>16	0.12 to >16
C419T^*g*^ (2)	3	4	2 to 4	>16	>16	>16 to >16
Silent mutations						
A120G^*h*^ (2)	2.25	4	0.5 to 4	>16	>16	>16 to >16
T159C^*i*^ (23)	0.25	4	0.06 to 4	4	>16	0.12 to >16
G168C^*j*^ (2)	0.625	1	0.25 to 1	>16	>16	0.5 to >16
G255A^*k*^ (10)	3	4	0.06 to 4	>16	>16	>16 to >16
G279T^*l*^ (9)	2	4	0.06 to 4	>16	>16	>16 to >16
A330C^*m*^ (22)	0.375	4	0.06 to 4	>16	>16	0.12 to >16
T336C^*n*^ (9)	2	4	0.06 to 4	>16	>16	>16 to >16
*rrl* mutation A2059G^*o*^ (1)			>16			>16

a IRT, initial reading time; LRT, late reading time.

b MIS128.

c MIS003, MIS034, MIS122, MIS124, MIS181, MIS194, MIS205, MIS300, MIS314, MIS317, and MIS354.

d MIS114.

e MIS219.

f MIS003, MIS005, MIS034, MIS114, MIS122, MIS124, MIS166, MIS181, MIS194, MIS205, MIS261, MIS283, MIS300, MIS311, MIS314, MIS317, MIS318, MIS324, MIS328, MIS334, and MIS354.

g MIS114 and MIS339.

h MIS166 and MIS283.

i MIS003, MIS005, MIS034, MIS114, MIS122, MIS124, MIS128, MIS166, MIS181, MIS194, MIS205, MIS261, MIS283, MIS300, MIS311, MIS314, MIS317, MIS318, MIS324, MIS328, MIS334, MIS339, and MIS354.

j MIS219 and MIS303.

k MIS005, MIS114, MIS166, MIS261, MIS283, MIS311, MIS318, MIS324, MIS328, and MIS334.

l MIS005, MIS114, MIS166, MIS261, MIS283, MIS311, MIS318, MIS324, and MIS328.

m MIS003, MIS005, MIS034, MIS114, MIS122, MIS124, MIS166, MIS181, MIS194, MIS205, MIS261, MIS283, MIS300, MIS311, MIS314, MIS317, MIS318, MIS324, MIS328, MIS334, MIS339, and MIS354.

n MIS005, MIS114, MIS166, MIS261, MIS283, MIS311, MIS318, MIS324, MIS328.

o MIS127.

### Partial *rpoB*, *hsp65*, *secA1*, *rrl*, and full *erm*(41) gene sequencing analyses.

Phylogenetic analyses of *erm*(41), *rpoB*, *hsp65*, and *secA1* of the 75 isolates identified them as MABC and classified them into three subspecies: 35 isolates of M. abscessus subsp. *abscessus* (35/75 [46.7%]), 39 of M. abscessus subsp. *massiliense* (39/75 [52%]), and 1 of M. abscessus subsp. *bolletii* (1/75 [1.3%]). There were 11 C28 sequevars and 24 T28 sequevars of *erm*(41) among the 35 M. abscessus subsp. *abscessus* isolates. Each of the 39 M. abscessus subsp. *massiliense* isolates contained a truncated *erm*(41) gene. The *erm*(41) T28 sequence was functional in the M. abscessus subsp. *bolletii* strain. Furthermore, there were no point mutations in gene *rrl* among the 35 M. abscessus subsp. *abscessus* isolates and 1 M. abscessus subsp. *bolletii* isolate. One of the 39 M. abscessus subsp. *massiliense* isolates (MIS127) contained an A2059G point mutation.

### Phylogenetic analysis.

**FIG 1:**
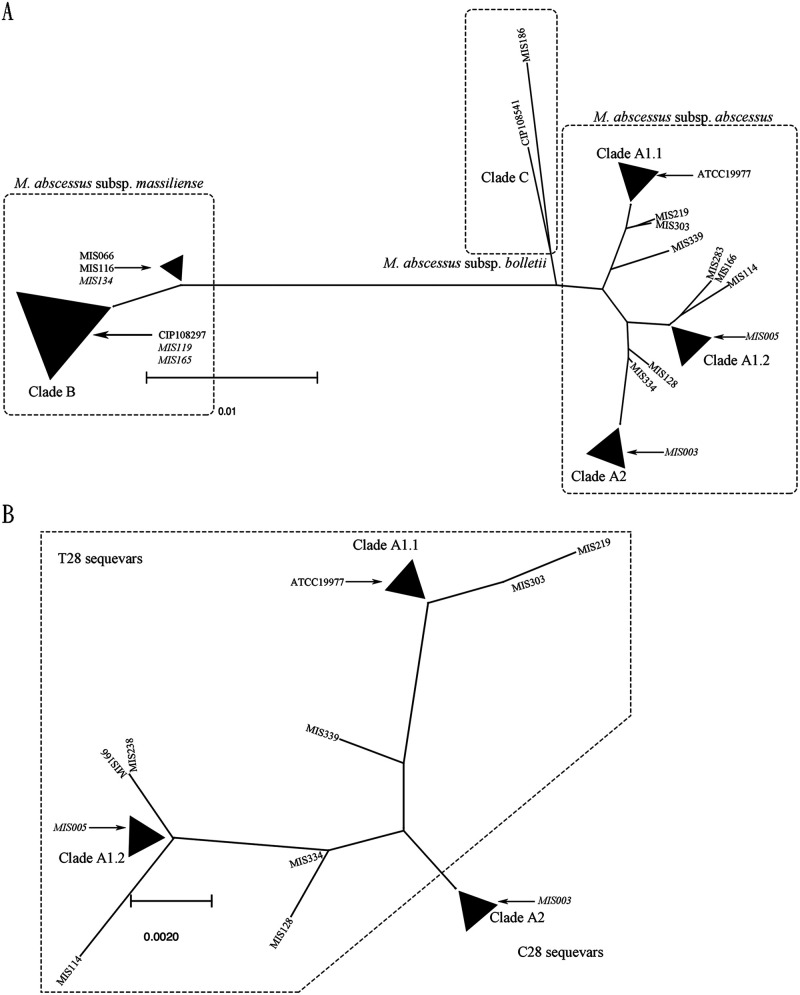
(A) Phylogenetic tree of the *erm*(41) gene of Mycobacterium abscessus species. Mycobacterium abscessus subsp. *abscessus* is identified through phylogenetic analysis of *erm*(41). *M*. *abscessus* subsp. *abscessus* (clades A1.1, A1.2, and A2) and Mycobacterium abscessus subsp. *bolletii* (clade C) have a closer genetic relationship with Mycobacterium abscessus subsp. *massiliense* [in the *erm*(41)] (clade B). Mycobacterium abscessus subsp. *bolletii* and most *M*. *abscessus* subsp. *abscessus* isolates possess an active *erm*(41) gene and inducible macrolide resistance. M. abscessus subsp. *massiliense*, however, has a truncated *erm*(41) gene, which cannot confer inducible macrolide resistance. Clade A1.1 (T28 sequevars): ATCC 19977, MIS032, MIS050, MIS102, MIS146, MIS179, MIS207, MIS221, MIS291, MIS297, and MIS299. Clade A1.2 (T28 sequevars): MIS005, MIS261, MIS311, MIS318, MIS324, and MIS328. Clade A2 (C28 sequevars): MIS003, MIS034, MIS122, MIS124, MIS181, MIS194, MIS205, MIS300, MIS314, MIS317, and MIS354. Clade B: CIP108297, MIS001, MIS007, MIS009, MIS039, MIS053, MIS063, MIS068, MIS070, MIS082, MIS085, MIS088, MIS100, MIS117, MIS119, MIS127, MIS131, MIS142, MIS147, MIS154, MIS165, MIS177, MIS180, MIS193, MIS203, MIS216, MIS238, MIS247, MIS251, MIS256, MIS288, MIS323, MIS325, MIS327, MIS337, MIS340, and MIS345. Clade C: CIP108541 and MIS186. Genetic mosaic strains are presented in italic. (B) Phylogenetic tree of the *erm*(41) gene of *M*. *abscessus* subsp. *abscessus*. Phylogenetic analysis of the *erm*(41) gene of M. abscessus subsp. *abscessus* demonstrates that C28 sequevars have less genetic diversity than T28 sequevars. The two exceptions are MIS128 and MIS219, which lack inducible macrolide resistance despite possessing the T28 sequevar of the *erm*(41) gene. Both have increased genetic diversity compared to other T28 sequevars in the *erm*(41) gene. Panel B depicts six additional *M*. *abscessus* subsp. *abscessus* T28 *erm*(41) outlines: MIS114, MIS166, MIS283, MIS303, MIS334, and MIS339. Clade A1.1 (T28 sequevars): ATCC 19977, MIS032, MIS050, MIS102, MIS146, MIS179, MIS207, MIS221, MIS291, MIS297, and MIS299. Clade A1.2 (T28 sequevars): MIS005, MIS261, MIS311, MIS318, MIS324, and MIS328. Clade A2 (C28 sequevars): MIS003, MIS034, MIS122, MIS124, MIS181, MIS194, MIS205, MIS300, MIS314, MIS317, and MIS354. Genetic mosaic strains are presented in italic.

In Fig. 1A, phylogenetic analysis of the *erm*(41) of MABC reveals that M. abscessus subsp. *abscessus* and M. abscessus subsp. *bolletii* are more closely related genetically than M. abscessus subsp. *massiliense*. *erm*(41) diversity was greater in M. abscessus subsp. *abscessus* subspecies than in the M. abscessus subsp. *massiliense* subspecies. The M. abscessus subsp. *abscessus* T28 and C28 sequevars were clearly distinguished in Fig. 1B. In the phylogenetic analysis of gene *erm*(41) of the M. abscessus subsp. *abscessus* subspecies (Fig. 1B), C28 sequevars exhibited more homogenous gene diversity, whereas T28 sequevars exhibited greater *erm*(41) heterogeneity. MIS128 and MIS219, which lacked inducible macrolide resistance, were outliers in the *erm*(41) phylogenetic tree from the branch of T28 sequevars (clades A2 and A1.1). The other six M. abscessus subsp. *abscessus* T28 *erm*(41) outliers (MIS114, MIS166, MIS283, MIS303, MIS334, and MIS339) from the clade A1.1, A1.2, and A2 branches, which contained inducible macrolide resistance, were T28 sequevars and are depicted in Fig. 1B. They possessed several silent mutations in the *erm*(41) gene, with the exception of MIS114’s G76A missense point mutation and MIS114’s and MIS339’s C419T missense point mutations.

**FIG 2:**
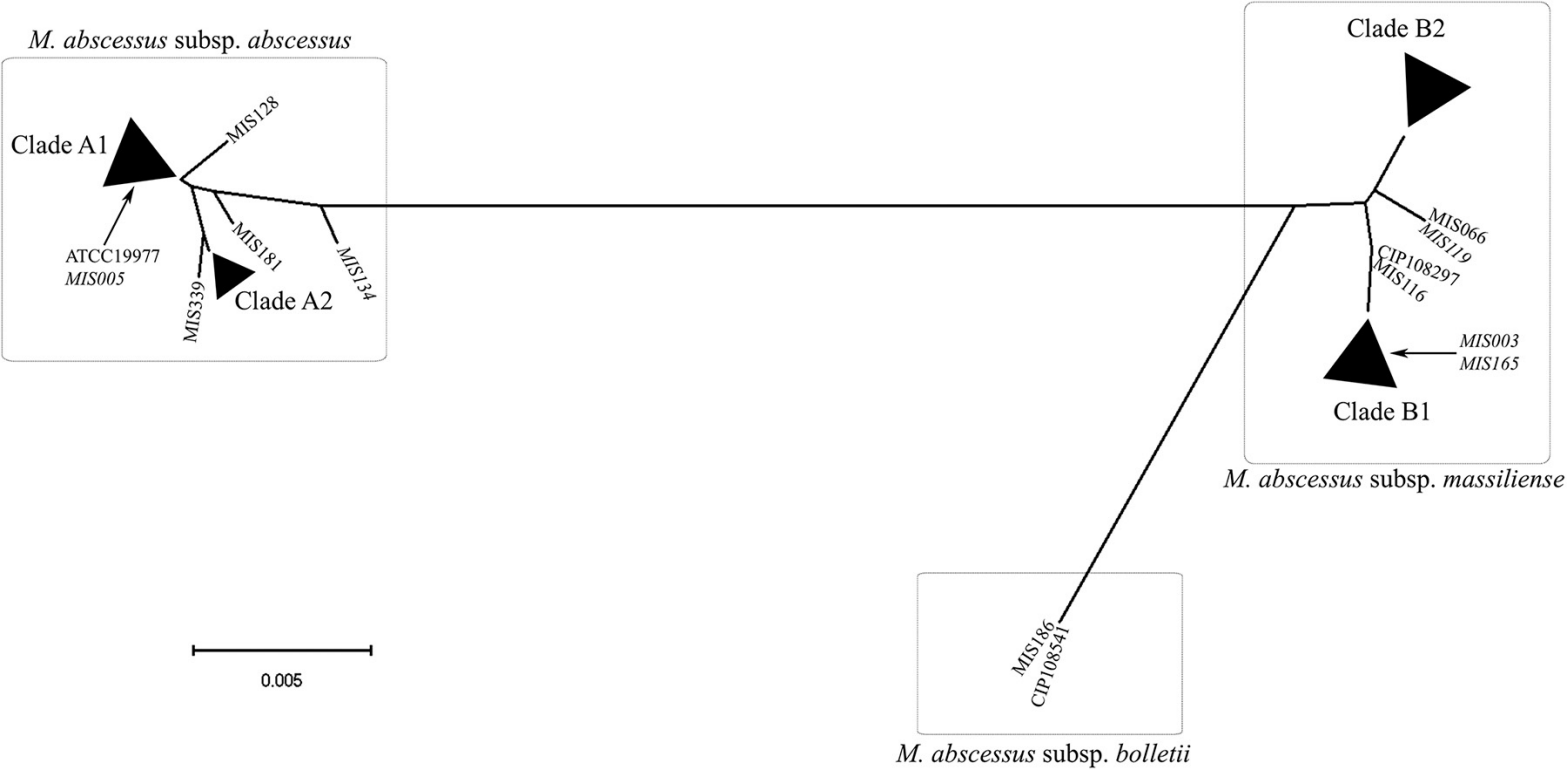
Phylogenetic tree of *rpoB* in Mycobacterium abscessus species. Analysis of the partial *rpoB* gene of Mycobacterium abscessus species reveals that M. abscessus subsp. *massiliense* and Mycobacterium abscessus subsp. *bolletii* share a closer genetic relationship in the *rpoB* gene than *M*. *abscessus* subsp. *abscessus*. In this figure, two isolates, MIS134 and MIS003, have a horizontal genetic transfer. NCBI BLAST database comparison and identification determined that isolate MIS134 is a member of the M. abscessus subsp. *massiliense* subspecies but possesses the *M*. abscessus subsp. *abscessus rpoB* gene. Isolate MIS003 is classified as *M*. abscessus subsp. *abscessus* but possesses the M. abscessus subsp. *massiliense rpoB* gene. The *rpoB* genes in MIS128 and MIS219 did not differ significantly from the other *M*. abscessus subsp. *abscessus rpoB* gene. Clade A1: ATCC 19977, MIS005, MIS032, MIS034, MIS050, MIS102, MIS114, MIS122, MIS124, MIS146, MIS166, MIS179, MIS194, MIS205, MIS207, MIS221, MIS261, MIS283, MIS291, MIS297, MIS299, MIS300, MIS303, MIS314, MIS317, MIS324, and MIS328. Clade A2: MIS219, MIS311, MIS318, MIS334, and MIS354. Clade B1: MIS003, MIS007, MIS039, MIS070, MIS082, MIS088, MIS142, MIS154, MIS165, MIS180, MIS203, MIS238, and MIS345. Clade B2: MIS001, MIS009, MIS053, MIS063, MIS068, MIS085, MIS100, MIS117, MIS127, MIS131, MIS147, MIS177, MIS193, MIS216, MIS247, MIS251, MIS256, MIS288, MIS323, MIS325, MIS327, MIS337, and MIS340. Genetic mosaic strains are presented in italic.

In Fig. 2, phylogenetic analysis of the partial *rpoB* gene of MABC revealed that the genetic relationship between M. abscessus subsp. *massiliense* and M. abscessus subsp. *bolletii* was the closest among the three subspecies of MABC. The phylogenetic analysis of *rpoB* did not reveal any significant differences between MIS128 and MIS219 and the other M. abscessus subsp. *abscessus* isolates.

**FIG 3:**
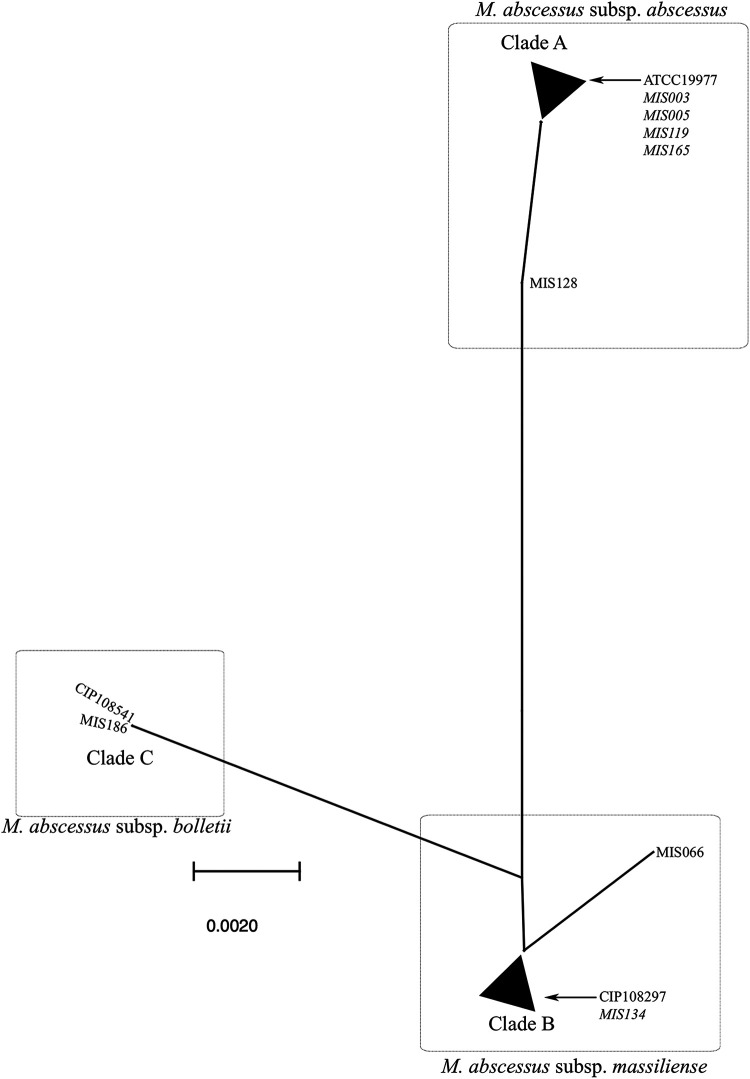
Phylogenetic tree of the *hsp65* gene of Mycobacterium abscessus species. Phylogenetic analysis of the partial *hsp65* gene of Mycobacterium abscessus species reveals that M. abscessus subsp. *massiliense* and Mycobacterium abscessus subsp. *bolletii* are distinct from *M*. abscessus subsp. *abscessus* in the *hsp65* gene, which is a reliable target for distinguishing *M*. abscessus subsp. *abscessus* from the other two subspecies. The NCBI BLAST database classifies MIS119 and MIS165 as M. abscessus subsp. *massiliense* subspecies, but they possess the *M*. abscessus subsp. *abscessus hsp65* gene due to horizontal gene transfer. Not only does isolate MIS128 have greater genetic diversity in the *erm*(41) gene than other *M*. abscessus subsp. *abscessus* isolates, but its *hsp65* gene polymorphism is distinct from those of other *M*. *abscessus* subsp. *abscessus* isolates. Comparing isolate MIS219 to other *M*. *abscessus* subsp. *abscessus*, increased genetic diversity was observed in the *erm*(41) gene but not in the *hsp65* gene. Clade A: ATCC 19977, MIS003, MIS005, MIS032, MIS034, MIS050, MIS102, MIS114, MIS119, MIS122, MIS124, MIS146, MIS165, MIS166, MIS179, MIS181, MIS194, MIS205, MIS207, MIS219, MIS221, MIS261, MIS283, MIS291, MIS297, MIS299, MIS300, MIS303, MIS311, MIS314, MIS317, MIS318, MIS324, MIS328, MIS334, MIS339, and MIS354. Clade B: CIP108297, MIS001, MIS007, MIS009, MIS039, MIS053, MIS063, MIS068, MIS070, MIS082, MIS085, MIS088, MIS100, MIS116, MIS117, MIS127, MIS131, MIS134, MIS142, MIS147, MIS154, MIS177, MIS180, MIS193, MIS203, MIS216, MIS238, MIS247, MIS251, MIS256, MIS288, MIS323, MIS325, MIS327, MIS337, MIS340, and MIS345. Clade C: CIP108541 and MIS186. Genetic mosaic strains are presented in italic.

Phylogenetic analysis of *hsp65* has proven to be a reliable target for distinguishing M. abscessus subsp. *abscessus* from the two other subspecies of MABC (16). In Fig. 3, the phylogenetic analysis of the partial *hsp65* gene of MABC revealed that M. abscessus subsp. *massiliense* and M. abscessus subsp. *bolletii* are distinct from M. abscessus subsp. *abscessus*. MIS219 demonstrated greater genetic diversity in *erm*(41) than in *hsp65* relative to other M. abscessus subsp. *abscessus* isolates. In contrast, MIS128 exhibited genetic diversity in *erm*(41) and polymorphism in *hsp65* that was distinct from that of the other M. abscessus subsp. *abscessus* isolates.

**FIG 4:**
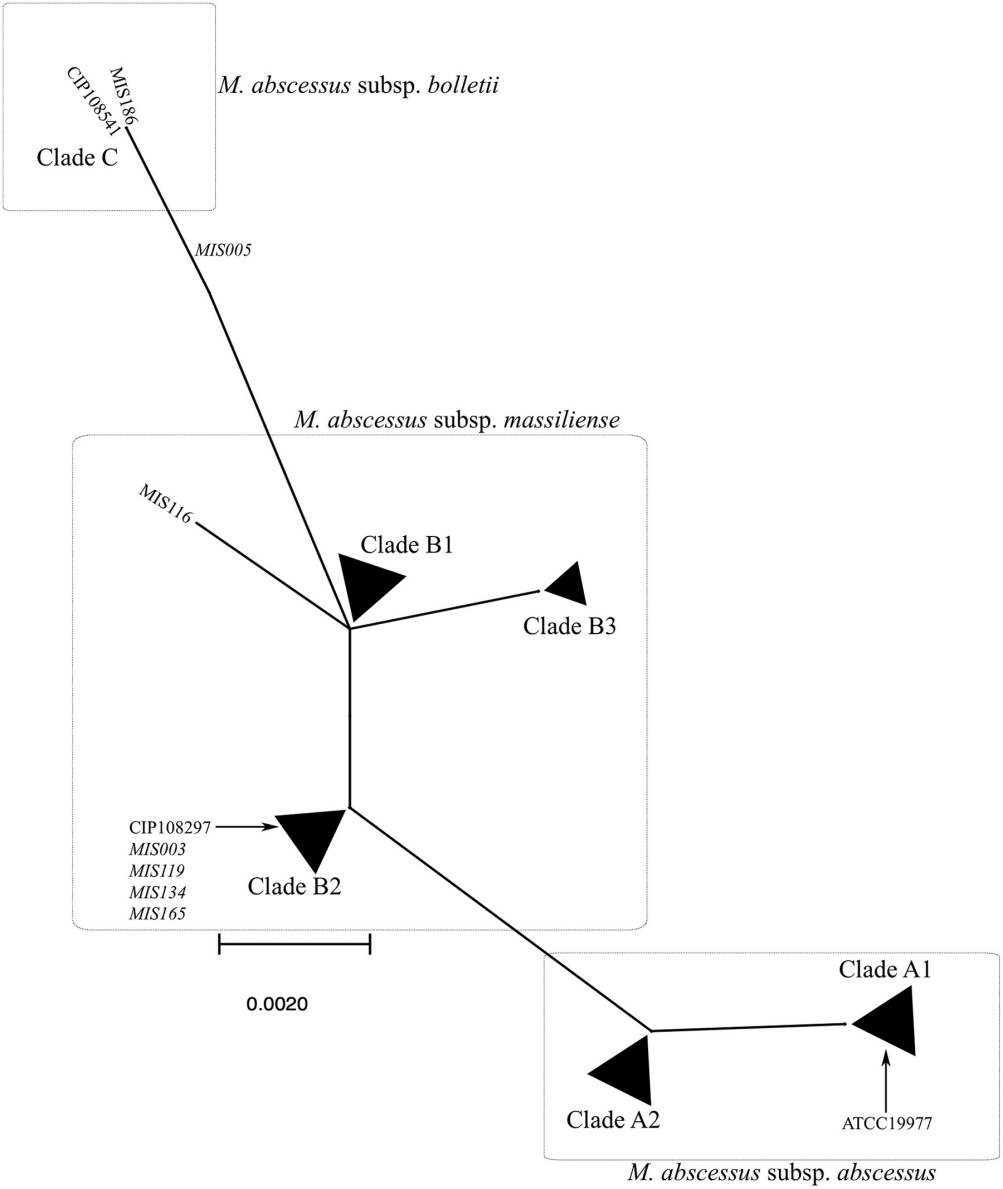
Phylogenetic tree of the *secA1* gene of Mycobacterium abscessus species. Phylogenetic analysis of the partial *secA1* gene of the Mycobacterium abscessus species reveals three subspecies that are genetically distinct from one another. BLAST analysis identifies the MIS005 isolate as belonging to *M*. abscessus subsp. *abscessus*, but it carries the *secA1* gene of Mycobacterium abscessus subsp. *bolletii*. In our study, horizontal gene transfer is observed not only between *M*. abscessus subsp. *abscessus* and M. abscessus subsp. *massiliense* but also between *M*. abscessus subsp. *abscessus* and M. abscessus subsp. *bolletii*. *M*. abscessus subsp. *abscessus* has a relatively conserved *secA1* gene compared to M. abscessus subsp. *massiliense*. Isolates MIS219 and MIS128 exhibited increased genetic diversity in the *erm*(41) gene but not in the *secA1* gene. Clade A1: ATCC 19977, MIS032, MIS034, MIS050, MIS102, MIS114, MIS122, MIS124, MIS146, MIS179, MIS181, MIS194, MIS207, MIS219, MIS221, MIS261, MIS291, MIS297, MIS299, MIS311, MIS314, MIS317, MIS324, MIS328, and MIS339. Clade A2: MIS128, MIS166, MIS205, MIS283, MIS300, MIS303, MIS318, MIS334, and MIS354. Clade B1: MIS001, MIS009, MIS053, MIS063, MIS066, MIS068, MIS085, MIS117, MIS131, MIS147, MIS216, MIS247, MIS251, MIS256, MIS288, MIS323, MIS325, MIS327, MIS337, and MIS340. Clade B2: CIP108297, MIS003, MIS007, MIS039, MIS070, MIS082, MIS088, MIS119, MIS134, MIS142, MIS154, MIS165, MIS180, MIS203, MIS238, and MIS345. Clade B3: MIS100, MIS127, MIS177, and MIS193. Clade C: CIP108541, MIS186, and MIS005. Genetic mosaic strains are presented in italic.

In Fig. 4, phylogenetic analysis of the partial *secA1* gene of MABC revealed the separation of three subspecies, which was useful for identifying MABC subspecies. In our study, *secA1* was more genetically diverse in M. abscessus subsp. *massiliense* than in M. abscessus subsp. *abscessus*. MIS128 and MIS219, the two outliers from the branches of *erm*(41) phylogenetic tree, demonstrated no increase in *secA1* genetic diversity among the M. abscessus subsp. *abscessus* isolates. Phylogenetic figures with a circular shape are displayed in Fig. S1 to S4 in the supplemental material.

### Genetic mosaicism.

The 75 isolates were divided into three subspecies based on *rpoB*, *hsp65*, *secA1*, and *erm*(41) gene sequencing and AST results. However, MIS005 was classified as M. abscessus subsp. *abscessus*. Using *secA1* gene sequencing and the BLAST database, MIS005 was identified as M. abscessus subsp. *bolletii*. Another isolate, MIS003, was identified as M. abscessus subsp. *abscessus* using *hsp65* and *erm*(41) gene sequencing, whereas *rpoB* and *secA1* sequencing revealed genetic mosaicism from the M. abscessus subsp. *massiliense* origin.

MIS134 was classified as M. abscessus subsp. *massiliense* based on the sequencing of its *hsp65*, *secA1*, and *erm*(41) genes using the NCBI BLAST database; however, it contained an M. abscessus subsp. *abscessus*-derived *rpoB* gene. MIS119 and MIS165 were identified as M. abscessus subsp. *massiliense* by *rpoB*, *secA1*, and *erm*(41) gene sequencing but exhibited a M. abscessus subsp. *abscessus hsp65* genotype.

## DISCUSSION

Nontuberculous mycobacterial (NTM) infections are on the rise globally, posing a grave threat to public health. MABC is recognized as the opportunistic pathogen with the highest pathogenicity among RGM. The treatment of MABC infection is difficult due to multidrug resistance and the lack of a prescription guideline for standard antimycobacterial agents for skin and soft tissue infections (10). Although the macrolide-containing regimen remained a cornerstone of anti-MABC therapy recommended by the international guidelines for both pulmonary and extrapulmonary infections (11), most MABC isolates possess inducible and/or acquired macrolide resistance, rendering macrolides less effective or ineffective. AMK and TGC were the most effective drugs against MABC in our study. As previously reported by Nash and colleagues and Brown-Elliott et al., CLA exhibited favorable activity against M. abscessus subsp. *massiliense* and C28 sequevars as well as some T28 sequevars of M. abscessus subsp. *abscessus* (15, 16). Similar to other reports in Taiwan, more than half of MABC strains had intermediate MICs to FOX and IMI. Other antimicrobial agents, such as quinolones, tetracyclines, LZD, and SXT, also demonstrated decreased activity in our study (5, 17). The relationship between the *erm*(41) and *rrl* genes and MABC’s macrolide resistance must be investigated.

Identification of the gene sequevars of *erm*(41) in MABC subspecies is essential for determining CLA susceptibility patterns. Nash et al. discovered in 2009 that MABC could produce an inducible 23S rRNA methylase that was encoded by *erm*(41) (16). Nash determined that the strains that appeared susceptible to CLA after 3 days of incubation became resistant to CLA after 14 days of incubation. Some isolates developed resistance to macrolides prior to 14 days. M. abscessus subsp. *abscessus* isolates contain an intact *erm*(41) gene, but they are separated into two *erm*(41) sequevars, distinguished by a T or C polymorphism at nucleotide 28. Typically, sequevar T28 isolates exhibit inducible resistance. In contrast, sequevar C28 isolates continue to be susceptible to CLA even after prolonged incubation. These results were consistent with those of numerous international studies (5, 15, 18, 19). In contrast, two M. abscessus subsp. *abscessus* T28 sequevars (MIS128 and MIS219) exhibited no inducible macrolide resistance and were susceptible to CLA. The M. abscessus subsp. *abscessus* clinical isolate MIS128 had a point mutation at codon 67 of the *erm*(41) gene, resulting in a stop codon instead of arginine. The nonsense mutation produced an incomplete, nonfunctional erythromycin ribosome methylase (Erm), which eliminated inducible macrolide resistance. Kim et al. (2016) reported a similar result (20). In their research, they discovered an isolate (SMC-Mabs-T19) with a C19T mutation that rendered it susceptible to CLA, despite being a M. abscessus subsp. *abscessus* T28 sequevar. The mutation resulted in a stop codon at codon 7 of the *erm*(41) gene, rendering *erm* inactive. Intriguingly, the *erm*(41) sequence of isolate MIS219 was different from the reference nucleotide sequences of *erm*(41) of M. abscessus ATCC 19977 by a single base, a G158A point mutation, resulting in a Gly53Asp missense mutation instead of a stop codon. The other missense point mutations were G76A, A238G, and C419T, which corresponded to the amino acid substitutions Glu26Lys, Ile80Val, and Pro140Leu. Further investigation requires further investigation to determine whether the single amino acid change affects the structure of the Erm protein. In addition to the nonsense point mutation and missense point mutations, Table 2 displays a number of silent point mutations, including A120G, T159C, G168C, G255A, G279T, A330C, and T336C. Silent mutation of DNA nucleotides, by definition, does not alter the amino acid sequences of the encoded proteins, but it does affect the RNA nucleotides of the corresponding mRNA. Changes in mRNA nucleotides could affect the secondary structure, mRNA stability, protein translation rate, protein folding, and posttranslational modifications of nascent polypeptide chains (21). Moreover, the presence of a highly impermeable cell envelope, multidrug efflux systems, and the production of several antibiotic-modifying/inactivating enzymes all contribute to the multidrug resistance of M. abscessus subsp. *abscessus* (9, 22). Understanding the exact mechanisms that render a T28 sequevar without macrolide resistance but with functional *erm*(41) could provide us with more information about the regulation of the *erm*(41) gene in MABC, which could aid in the development of new macrolide derivatives capable of overcoming inducible macrolide resistance and *rrl* hot spot point mutations.

M. abscessus subsp. *abscessus* isolates were distributed into three major *erm*(41) phylogenetic clades, including clades A1.1, A1.2, and A2. There were three phylogenetic outliers (MIS219, MIS303, and MIS339) from the branch of clade A1.1, 3 phylogenetic outliers (MIS114, MIS166, and MIS283) from the branch of clade A1.2, and 2 phylogenetic outliers (MIS128 and MIS334) from the branch of clade A2 (Fig. 1A). Several silent point mutations (A120G, T159C, G168C, G255A, G279T, A330C, and T336C), some missense point mutations (G76A, G158A, A238G, and C419T), and one nonsense point mutation (C199T) were found in these isolates as shown in Table 2. The MIS128 isolate possessed a nonsense point mutation (C199T) that corresponds to mutation of an arginine to a stop codon. Another strain, MIS219, possessed a unique missense point mutation (G158A) that corresponds to Gly53Asp. We discovered that not all M. abscessus subsp. *abscessus* T28 *erm*(41) phylogenetic outliers lost inducible macrolide resistance. However, all T28 sequevars lacking inducible macrolide resistance are *erm*(41) phylogenetic outliers.

Five of 75 MABC isolates showed genetic mosaicism of the *erm*(41), *rpoB*, *hsp65*, and *secA1* genes. Isolate MIS005 was a T28 M. abscessus subsp. *abscessus* sequevar with a functional *erm*(41) gene that conferred inducible macrolide resistance and a horizontal gene transfer from the M. abscessus subsp. *bolletii secA1* gene. We tentatively christened it M. abscessus subsp. *abscessus* hybrid *bolletii*. As with the other C28 sequevars, MIS003, a C28 M. abscessus subsp. *abscessus* sequevar with M. abscessus subsp. *abscessus*-derived *hsp65* and M. abscessus subsp. *massiliense*-origin *rpoB* and *secA1* genes, was susceptible to CLA IRT and LRT. We tentatively christened it M. abscessus subsp. *abscessus* hybrid *massiliense*. By sequencing the *erm*(41), *hsp65*, and *secA1* genes, isolate MIS134 was determined to be M. abscessus subsp. *massiliense*, whereas *rpoB* was transferred from M. abscessus subsp. *abscessus*. By sequencing the *erm*(41), *rpoB*, and *secA1 genes*, MIS119 and MIS165 were identified as M. abscessus subsp. *massiliense* isolates, whereas *hsp65* was transferred from M. abscessus subsp. *abscessus*. We provisionally designated the aforementioned three isolates as M. abscessus subsp. *massiliense* hybrid *abscessus*. All three M. abscessus subsp. *massiliense* isolates had a truncated *erm*(41) gene and exhibited the same susceptibility to CLA as other M. abscessus subsp. *massiliense* isolates. Although many M. abscessus subsp. *abscessus* and M. abscessus subsp. *massiliense* isolates exhibited genetic mosaicism for *rpoB*, *hsp65*, and *secA1* in this study, they exhibited the same macrolide susceptibility as other *erm*(41) sequevars. The evolutionary significance of genetic mosaicism is to be determined (23).

All subspecies of MABC were susceptible to AMK and TGC, which were previously recommended as part of the empirical antimicrobial regimens with/without CLA for MABC skin and soft tissue infections (10). The macrolide-susceptible M. abscessus subsp. *abscessus* T28 sequevars were outliers of the *erm*(41) phylogenetic branch, but not all outliers were macrolide susceptible. In addition, genetic mosaicism of *rpoB*, *hsp65*, and *secA1* among MABC isolates is not uncommon and should be taken into account when identifying the subspecies and developing therapeutic regimens for MABC that target more than 23S rRNA, such as glycylcyclines or new oxazolidinones. However, this is a single-center study with a limited number of clinical MABC strains. Strong recommendations require a larger sample size, additional multicenter studies, and more experiments on the structure of the Erm protein.

## MATERIALS AND METHODS

### Ethics statement.

The Chang Gung Medical Foundation Institutional Review Board reviewed and authorized the study’s objectives and procedures (no. 201601809B0).

### Clinical strains.

A total of 75 MABC clinical strains were isolated from patients with skin and soft tissue infections at Chang Gung Memorial Hospital, Linkou Medical Center, Taoyuan, Taiwan, from 1 August 2012 to 31 March 2018. They were stored in skim milk with 50% glycerol in a refrigerator at −70°C until they were utilized in experiments.

### Antimicrobial susceptibility testing.

When growth was optimal, bacterial isolates were subcultured on Middlebrook 7H11 agar plates and incubated at 30°C in room air for 3 to 5 days. The CLSI-recommended broth microdilution technique was utilized with Sensititre RAPMYCO MIC plates (Thermo Fisher, Cleveland, OH) (24). The drug concentration ranges of the 11 antimycobacterial agents of these plates were as follows: AMK, 1 to 64 μg/mL; FOX, 4 to 128 μg/mL; CIP, 0.12 to 4 μg/mL; CLA, 0.06 to 16 μg/mL; DOX, 0.12 to 16 μg/mL; IMI, 2 to 64 μg/mL; LZD, 1 to 32 μg/mL; MIN, 1 to 8 μg/mL; MXF, 0.25 to 8 μg/mL; TGC, 0.015 to 4 μg/mL; and SXT, 0.25/4.75 to 8/152 μg/mL. The MICs of CLA were read at an IRT (typically between the 3rd and 5th days of incubation) if control growth was positive and at an LRT (typically between the 10th and 14th days) to detect inducible macrolide resistance. The MIC breakpoints for susceptible, intermediate, and resistant organisms followed CLSI guidelines. The intermediate breakpoints of these antibiotics were those proposed by the CLSI, with AMK at 32 μg/mL, FOX at 32 to 64 μg/mL, CIP at 2 μg/mL, CLA at 4μg/mL, DOX at 2 to 4 μg/mL, IPM at 8 to 16 μg/mL, LZD at 16 μg/mL, meropenem (MPM) at 8 to 16 μg/mL, and MXF at 2 μg/mL. The MICs of Mycobacterium peregrinum ATCC 700686 were used as a quality control measure. According to CLSI recommendations, its acceptable MIC ranges are as follows: AMK, <1 to 4 μg/mL; FOX, 4 to 32 μg/mL; CIP, <0.12 to 0.5 μg/mL; CLA, <0.06 to 0.5 μg/mL; DOX, 0.12 to 0.5 μg/mL; IMI, 2 to 16 μg/mL; LZD, 1 to 8 μg/mL; MIN, 0.12 to 0.5 μg/mL; MXF, <0.06 to 0.25 μg/mL; and SXT, <0.25/4.8 to 2/38 μg/mL.

### *erm*(41) full-gene and partial *rpoB*, *hsp65*, *secA1*, and *rrl* gene sequencing.

Following the manufacturer’s instructions, 75 strains of bacterial genomic DNA were extracted using the High Pure viral nucleic acid kit (Roche, Mannheim, Germany). Subsequently, using the sequences of the three housekeeping genes *rpoB*, *hsp65*, and *secA1*, 75 MABC isolates were classified into subspecies. Prior to this, Zelazny et al. (25) described the specifics of the PCR method. The inducible and acquired macrolide resistance was determined by sequencing the *erm*(41) and partial *rrl* genes. Maurer et al. (26) have previously described the methods for *erm*(41) and *rrl* sequencing. The *rrl* sequencing corresponded to nucleotides 1978 through 2728 in the *rrl* of E. coli. Next, using the BLAST method, the sequences of these five genes from each isolate were examined and compared to sequences in the NCBI database (https://blast.ncbi.nlm.nih.gov/Blast.cgi). M. abscessus subsp. *abscessus* ATCC 19977, M. abscessus subsp. *massiliense* CIP108297, and M. abscessus subsp. *bolletii* CIP108541 were used as the type strains for gene sequencing and phylogenetic analysis.

### Phylogenetic analysis.

M. abscessus subsp. *abscessus*, M. abscessus subsp. *massiliense*, and M. abscessus subsp. *bolletii* isolates’ partial *rpoB*, *hsp65*, and *secA1* genes and full *erm*(41) genes were aligned and analyzed for phylogenetic trees using the MEGA X software (27). DNA sequences were aligned using multiple-sequence comparison with the log-expectation program (28), and the evolutionary history was inferred using the neighbor-joining method (29). The evolutionary distances were computed using the maximum composite likelihood method (30). All ambiguous positions were removed for each sequence pair (pairwise deletion option).

### Statistical analysis.

Stata 17 (College Station, TX: StataCorp LLC) was utilized as the statistical software. Intergroup MICs were compared by the Mann-Whitney *U* test. A *P* value of *<*0.05 was considered statistically significant.

### Data availability.

The representative 75 sequences of the MABC genes focused on in this study gene have been deposited in GenBank under the following accession numbers: *erm*(41), OP354418 through OP354452 and OP360947 through OP360986; *rpoB*, OP382275 through OP382349; *hsp65*, OP422734 through OP422808; *secA1*, OP422812 through OP422886; and 10 sequences representing each *rrl* gene, OP430560 through OP430569.
