# Supplementary material for: Impact on Macrolide Resistance of Genetic Diversity of Mycobacterium abscessus Species
Source: Microbiol Spectr. 2022 Nov 23;10(6):e02749-22. doi: 10.1128/spectrum.02749-22 (PMC9769998; doi:10.1128/spectrum.02749-22)

Supplemental Material

Supplementary Figure 1. Phylogenetic tree of the *erm*(41) gene of *Mycobacterium abscessus* species.

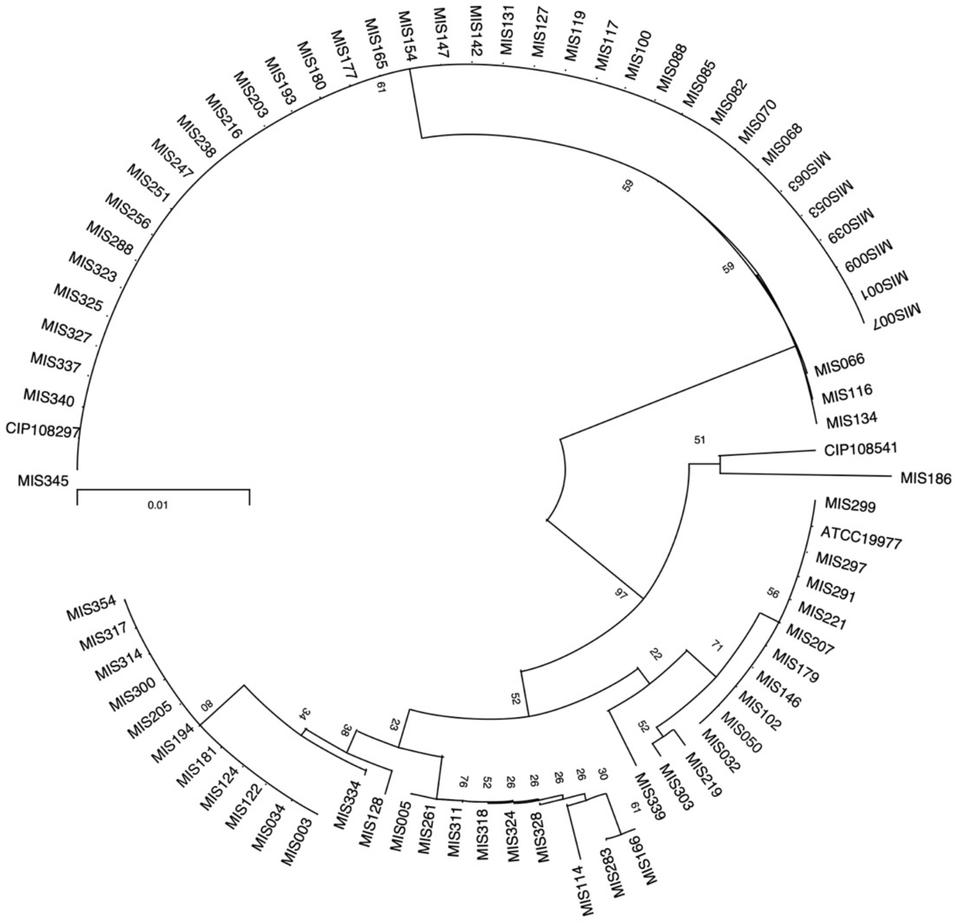

Supplementary Figure 2. Phylogenetic tree of the *rpoB* gene of *Mycobacterium abscessus* species.

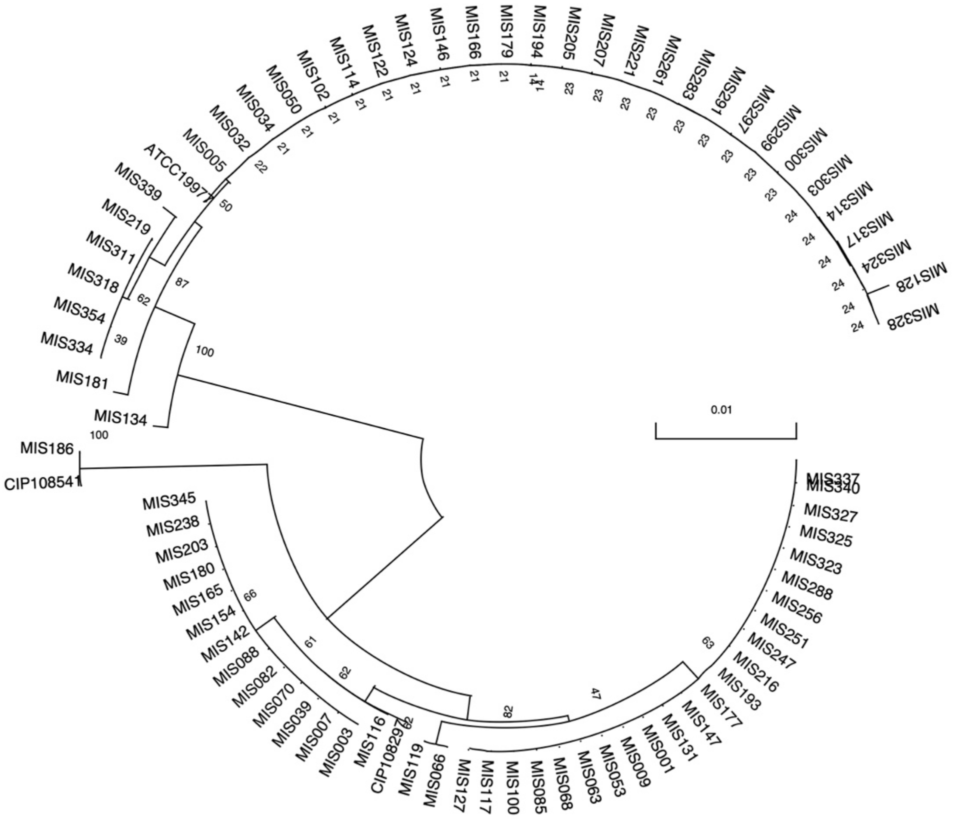

Supplementary Figure 3. Phylogenetic tree of the *hsp65* gene of *Mycobacterium abscessus* species.

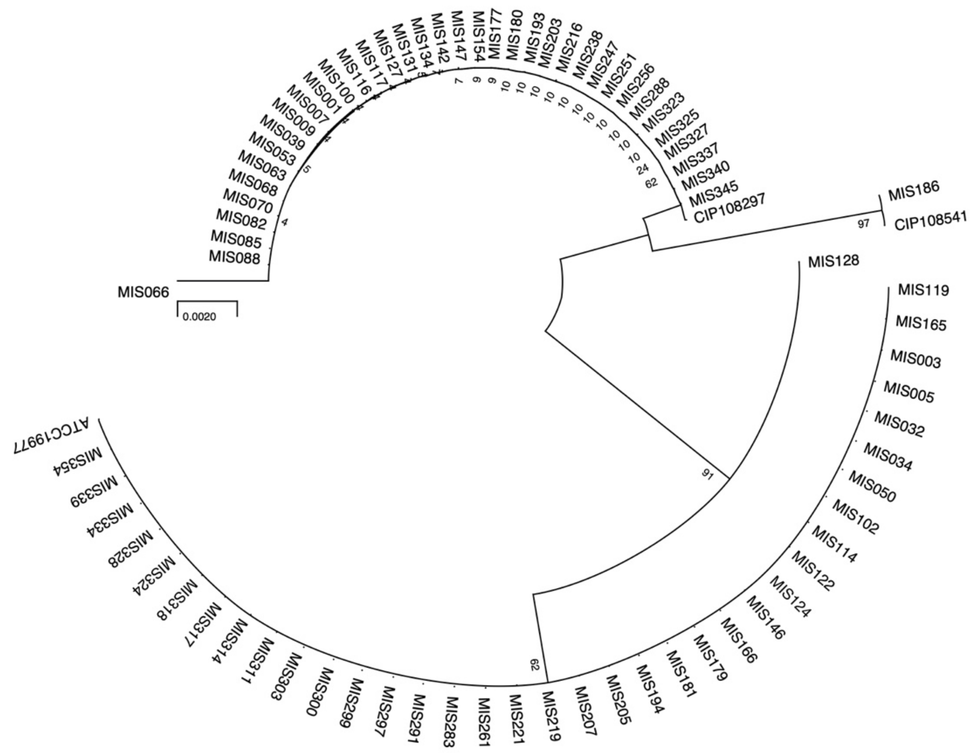

Supplementary Figure 4. Phylogenetic tree of the *secA1* gene of *Mycobacterium abscessus* species.

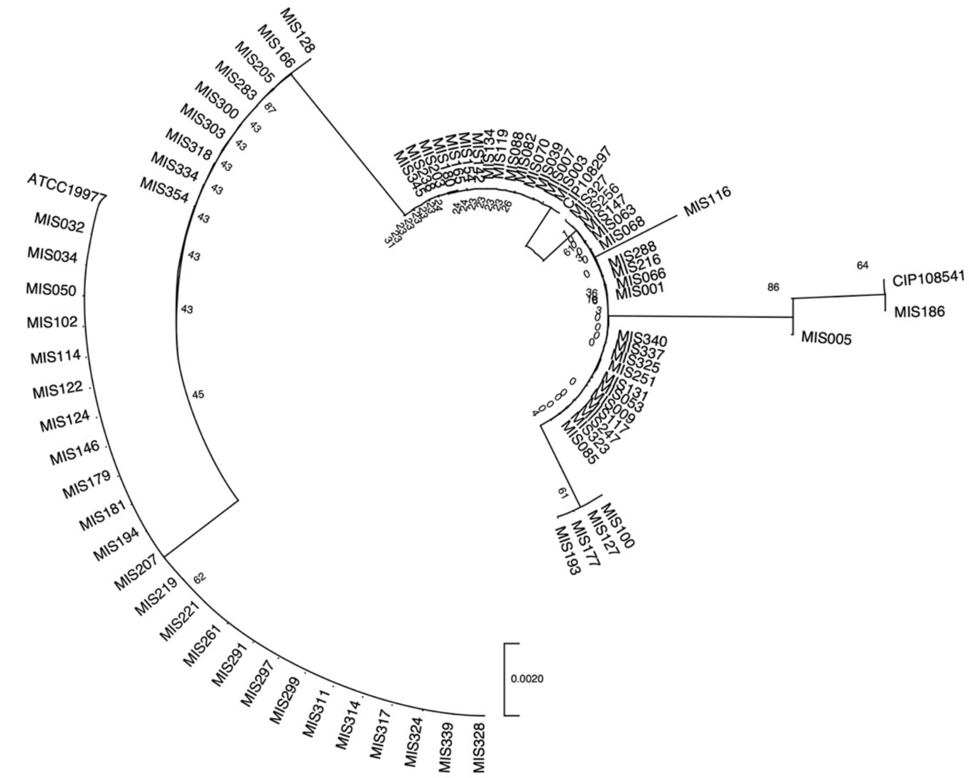

Supplement: Supplemental file 1 — Supplemental material. Download spectrum.02749-22-s0001.pdf, PDF file, 0.8 MB [file spectrum.02749-22-s0001.pdf]
